# Detection of Prion Infectivity in Fat Tissues of Scrapie-Infected Mice

**DOI:** 10.1371/journal.ppat.1000232

**Published:** 2008-12-05

**Authors:** Brent Race, Kimberly Meade-White, Michael B. A. Oldstone, Richard Race, Bruce Chesebro

**Affiliations:** 1 Laboratory of Persistent Virus Diseases, Rocky Mountain Laboratories, National Institute of Allergy and Infectious Diseases, National Institutes of Health, Hamilton, Montana, United States of America; 2 Department of Immunology and Microbial Science, The Scripps Research Institute, LaJolla, California, United States of America; University of Edinburgh, United Kingdom

## Abstract

Distribution of prion infectivity in organs and tissues is important in understanding prion disease pathogenesis and designing strategies to prevent prion infection in animals and humans. Transmission of prion disease from cattle to humans resulted in banning human consumption of ruminant nervous system and certain other tissues. In the present study, we surveyed tissue distribution of prion infectivity in mice with prion disease. We show for the first time detection of infectivity in white and brown fat. Since high amounts of ruminant fat are consumed by humans and also incorporated into animal feed, fat-containing tissues may pose a previously unappreciated hazard for spread of prion infection.

## Author Summary

Prion diseases, also known as transmissible spongiform encephalopathies, are infectious progressive fatal neurodegenerative diseases which affect humans as well as wild and domestic animals. Distribution of prion infectivity in organs and tissues is important in understanding prion disease pathogenesis and designing strategies to prevent prion infection in animals and humans. We show for the first time the presence of prion infectivity in white fat and brown fat tissues of mice with prion disease. Our results suggest that fat tissues of domestic or wild animals infected with prions may pose an unappreciated hazard for spread of infection to humans or domestic animals. The presence of prion infectivity in fat suggests that additional consideration may be required to eliminate from the food chain any fat from ruminants suspected of exposure to or infection with prions. Thus, this finding has implications for public health, food safety, and prion disease prevention strategies.

## Introduction

Prion diseases, also known as transmissible spongiform encephalopathies (TSE), are infectious progressive fatal neurodegenerative diseases which affect humans as well as wild and domestic animals [1]–[3]. Some examples include Creutzfeldt-Jakob disease (CJD) in humans, scrapie in sheep and goats, bovine spongiform encephalopathy (BSE) in cattle and chronic wasting disease (CWD) in North American deer and elk. Usually the infection shows a strong species-specificity, but cross-species infection can occur naturally or experimentally often at a low efficiency. The possibility that humans might contract prion disease by exposure to infected animals is a major public health concern. In the past decades transmission of BSE from cattle appears to account for the occurrence of variant CJD in humans. This has led to the necessity of identifying the tissues harboring infectivity in diseased animals.

Previously, infectivity has been surveyed in multiple organs of sheep [4],[5], goats [6], cattle [7],[8], deer [9], mink [10], mice [11],[12], and hamsters [13]. The highest levels of infectivity and protease-resistant prion protein (PrPres) were found in nervous system tissues such as brain, spinal cord and eye. In some animal species and prion strains, intermediate to low levels of infectivity and PrPres were found in spleen, lymph node, peripheral nerves and intestine. The lowest infectivity levels were found in a variety of organs including skeletal muscle, tongue, salivary gland, heart, liver, and kidney. It was also noteworthy that in BSE infected cattle, both spleen and skeletal muscle were negative or extremely low by infectivity testing, suggesting that BSE infected cattle differ from other models in this aspect [7],[8]. PrPres was also detected by western blot in tongue of hamsters and sheep [13]–[15], skeletal muscles of mice, sheep, and hamsters [12],[13],[16],[17] and cardiac muscle of deer and elk [18]. IHC analysis for PrPres in these tissues was also often positive in myocytes and nerves while fat tissue adjacent to muscle was not noted to be positive [13]–[21].

In some studies no infectivity was detected in blood and serum [4],[7],[11], but more recently low levels of blood infectivity have been detected using more sensitive methods [22]. Blood is also positive for PrPres using protein misfolding cyclic amplification (PMCA) [23], however this test does not directly measure infectivity.

We previously found a high level of infectivity in brain and heart of scrapie-infected tg44 transgenic mice expressing anchorless PrP [24], suggesting the possibility that these mice might have an increased infection at other extraneural sites [25]. In our preliminary histopathology studies of infected tg44 mice we noted the presence of PrPres in fat tissues (B. Chesebro, unpublished observations). Adipose tissue is widely consumed by humans and is also used in some animal feeds, but has not been specifically studied as a possible site of accumulation of prion infectivity. Therefore, in the present study we examined white and brown fat tissues as well as several other tissues of scrapie-infected WT and tg44 mice by quantitative end-point dilution infectivity titration as well as immunoblotting and IHC for presence of PrPres. White and brown fat tissues of both types of mice were found to contain significant scrapie infectivity, and thus appear to be a previously unrecognized site of prion accumulation in vivo.

## Results

### Infectivity Titers in Tissues of Scrapie-Infected WT and tg44 Mice

In the present paper we examined the tissue distribution of prion infectivity in scrapie-infected WT mice. Infectivity was detected in all tissues tested including some plasmas (Table 1). Brain titers were 8.3–9.3 log_10_ID_50_/gram, titers in skeletal muscle, tongue, white fat, brown fat and liver were from 3.6–5.2 log_10_ID_50_/gram, and very low titers were seen in plasma (2.9-<1.8 log_10_ID_50_/ml). To our knowledge this is the first report of infectivity in brown or white fat tissue of any species.

**Table 1:** Infectivity titers in scrapie-infected WT and anchorless PrP transgenic (tg44) mice.

	Anchorless PrP (tg44)		WT	
Tissue^a^	Titers^b^	Avg	Titers^b^	Avg
Brain	9.3, 10.3	9.8	9.0, 9.3, 8.3^e^	8.9
Skeletal muscle (quadriceps)	7.5, 9.3, 6.3^c^, 7.7^c^	7.7	3.8, 4.5, ≥5.8, ≥5.8, 6.3, 5.0^e^	5.2
Tongue	9.0, 9.3, 7.8^c^	8.7	5.5, 3.8, 3.8, 5.5, 5.0^e^	4.7
White fat (subcutaneous)	9.5, 7.8^c^	8.6	5.3, 5.3, 4.0, 3.8, 5.5, 4.3^e^	4.7
White fat (perirenal)	9.5, 7.5, 7.8^c^, 7.6^c^	8.1	<2.8, 3.3, 3.6, ≥4.8, 3.5, 5.2^e^	3.9
Brown fat	≥9.8, 9.3, 8.3^e^, 7.6^e^	8.7	5.3, 3.8, 3.8	4.3
Liver	5.5, 8.3, 6.1^c^, 5.4^c^	6.3	3.8, 3.3, 3.5, 3.3, 4.1, 3.9	3.6
Plasma^d^	5.3^c^, 4.1, 3.9, 3.5, 3.0^c^, 2.5^e^, <1.8^e^, 1.8^e^	3.2	2.9, 1.8, 2.1, <1.8, <1.8, <1.8^e^	1.8

a Following IC inoculation, tissues were analyzed at 482–654 days for tg 44 mice and 155–165 dpi for WT mice. Plasmas were analyzed from 365–596 dpi for tg44 and 155–165 dpi for WT mice.

b Titers are expressed as log_10_ID50/gram tissue or ml plasma [37]. When recipient mice were inoculated with 50 µl of a 1% tissue homogenate or 50 µl of a 1/100 plasma dilution, the lower limit of detection was 2.8 log_10_ID50/gram tissue or ml plasma. Some mice were inoculated with 50 µl of a 1/10 dilution of plasma, so the lower limit of detection was 1.8 log_10_ID50/ml plasma. Tissues from mock-inoculated or uninoculated mice had no detectable infectivity.

c Two tg44 mice inoculated IP were sacrificed at 441 and 692 dpi.

d These low or negative plasma titers differed from a previous report [25], where titers of >5.0 and >7.0 log_10_ID_50_/ml were found in two anchorless PrP tg mice at 480 and 507 days post-infection with the RML scrapie strain.

e Mice inoculated with RML scrapie strain.

Mice without footnote were inoculated with 22L scrapie strain.

We reported previously that scrapie-infected tg44 mice did not develop clinical signs over a 700 day period [24]. In the present study after scrapie infection of tg44 mice, high titers were found in brain, skeletal muscle, tongue, white fat and brown fat (Table 1). Plasma titers were 3.0–5.3 log_10_ID_50_/ml in mice infected with 22L scrapie, and ranged from 2.5 to<1.8 log_10_ID_50_/ml in mice infected with RML scrapie. These low plasma titers indicated that contamination by plasma could not account for the high level of infectivity found in other positive tissues described here.

### Detection of PrPres by Immunoblot or Immunohistochemistry

**Figure 1:**
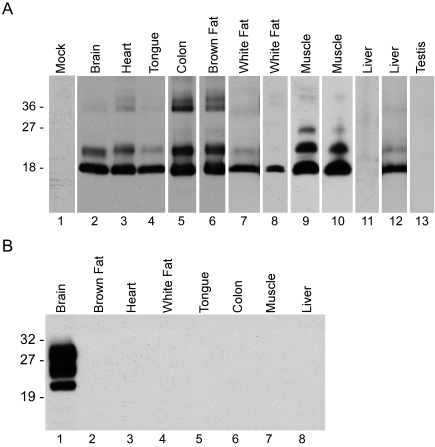
Immunoblot detection of PrPres from various tissues of 22L scrapie-infected anchorless PrP tg44 mice and WT mice. Blots were developed using D13 anti-PrP [26]. No PrPres was detected in uninoculated tg44 or WT mice. For tg44 mice (A), lane 1, mock infected tg44 mouse brain, 1.0 mg tissue equivalents. Lanes 2–6 were loaded with 0.25–0.5 mg tissue equivalents and developed using an enhanced chemiluminescence exposure of 5 seconds, and lanes 7–13 were loaded with 1.0 mg tissue equivalents and exposed for 1–2 minutes. Lane 11, liver was negative, and lane 12 was the only positive liver of nine tested. In tg44 mice, spinal cord, spleen, lymph node, intestines, kidney, and lung were also positive, and eye and skin were negative (data not shown). Note different PrPres banding pattern seen in tg44 mice: predominant band is the 18 kD unglycosylated form. Next in abundance is the 22kD monoglycosylated form. The 25kD diglycosylated form is barely visible in lanes 5, 6, 9, and 10. Dimeric PrP bands at 36kD are seen most clearly in lanes 3, 5, 6, and 9. For WT mice (B), lane 1 was loaded with 0.25 mg tissue equivalents while lanes 2–8 were loaded with 1.0 mg tissue equivalents. Blot was exposed for 2 minutes to search for possible weak bands in lanes 2–8. Lane 1 was overexposed, but showed usual PrPres banding pattern for 22L mouse scrapie brain. Results are representative of tissues from at least five mice analyzed in each group.

Because prion infectivity and PrPres are usually associated, we examined the tissues presented in Table 1 for PrPres detectable by immunoblot with antibody D13 [26]. In tissues of tg44 mice, PrPres was readily detected by standard immunoblot in brain, heart, tongue, colon and brown fat (Figure 1A). By loading an increased amount of tissue extract, PrPres was also detected in white fat, muscle and one liver (out of 9 tested), but not in testes (Figure 1A).

In infected WT mice, immunoblotting showed a strong PrPres signal from brain but the other seven tissues tested were negative (Figure 1B). To enhance the sensitivity of PrPres detection, fat tissues of WT mice were also tested by PTA immunoblot [27],[28]. Results were still negative, however using this method with fat tissues there were some technical problems which were not observed on positive control brain samples (data not shown).

**Figure 2:**
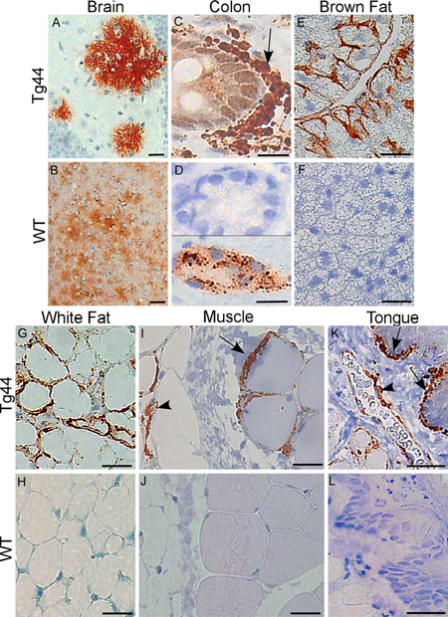
Immunohistochemical detection of PrPres in tissues of 22L infected tg44 and WT mice. Each tissue is presented as a pair, tg44 on top and WT on the bottom. Tissue sections were stained with D13 anti-PrP (red). (A,B) Brain. (C,D) Colon. The arrow in (C) shows extensive PrP deposition in the lamina propria. The red nuclear staining of the epithelial cells is an artifact. (D) Top, PrP-negative villi and lamina propria; bottom, PrP-positive enteric ganglion in the muscularis layer. (E,F) Brown fat. (G,H) White fat. (I,J) Muscle. In (I) the arrow indicates PrP accumulation on the surface of a myocyte. White fat adjacent to the muscle also shows PrP staining (arrowhead). (K,L) Tongue. In (K) the arrows indicate PrPres staining near the subepithelial layer of the tongue. The arrowhead shows PrPres adjacent to a blood vessel. Scale bars indicate 25 µm, except in (I,J) where bars are 12 µm.

**Figure 3:**
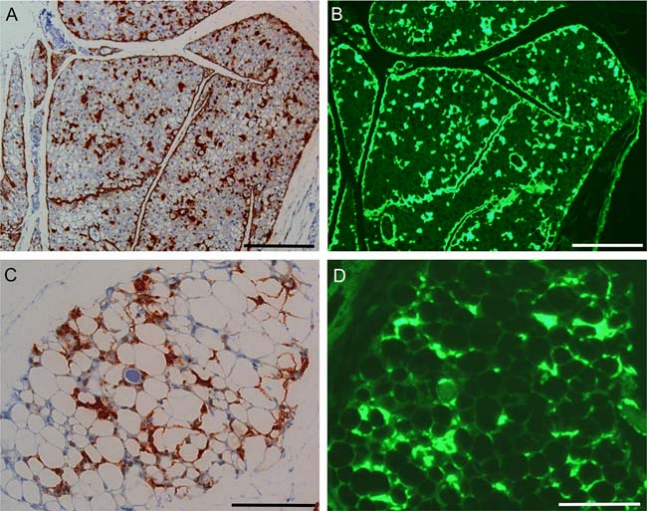
Amyloid PrPres in brown and white fat from a homozygous tg44 mouse. Tissues shown are from a tg44 mouse inoculated with 22L scrapie and euthanized at 350 days post infection. (A,B) Brown fat. (C,D) White fat. (A,C) Staining with anti-PrP antibody D13 shows a distinct perilobular and perivascular distribution of PrPres. (B,D) Amyloid staining with Thioflavin S shows a similar distribution pattern of amyloid.. (A,B) and (C,D) were approximately 12 micrometers from each other, respectively. Scale bar in (A,B) is 200 micrometers and 100 micrometers in (C,D).

Tissues were also examined by immunohistochemistry (IHC) for presence of PrPres (Figure 2). WT mice had detectable PrPres only in brain, neuronal ganglia of colon (Figure 2D, lower panel) and spleen (not shown). Lack of detectable PrPres in WT brown fat, white fat, muscle, tongue and liver appeared to be related to lower infectivity titers in these tissues compared to brain (Table 1). In contrast, in infected tg44 mice, extraneural tissues including lamina propria of colon, brown fat, white fat, muscle and tongue had PrPres detectable by IHC (Figure 2). Furthermore, this PrPres was stained by the amyloid stain, Thioflavin S, in both brown and white fat (Figure 3A–3D) and in other tissues (not shown). These IHC results were in agreement with the previous infectivity data (Table 1) and immunoblotting data (Figure 1A) and confirmed that there was a high level of extraneural prion disease infection in multiple tissues of tg44 mice.

## Discussion

**Figure 4:**
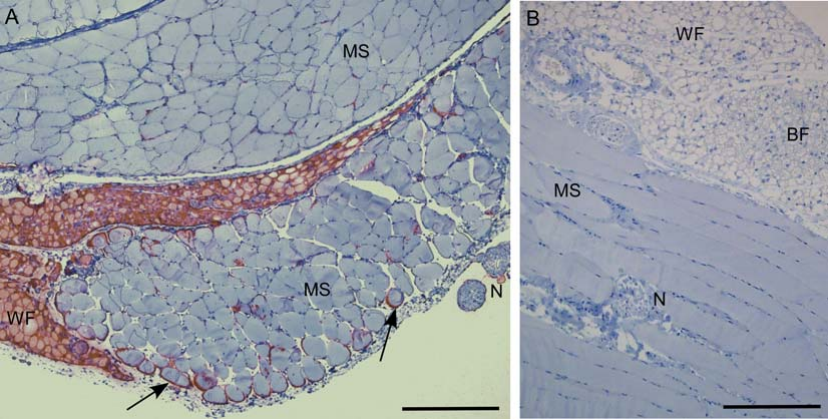
PrP in white fat and adjacent skeletal muscle. Detection of PrPres (red staining) used D13 anti-PrP antibody. (A) Myocytes (MS) adjacent to white fat (WF) of a homozygous tg44 mouse at 375 days post-infection with 22L scrapie. PrPres staining in fat was much more intense than in muscle (arrows), suggesting a lower level of infection in muscle. Nerves (N) had minimal staining. White fat showed smaller than usual lipid lobules, probably a consequence of clinical wasting. (B) Uninfected tg44 mouse. Fat, muscle, nerve, and brown fat (BF) are negative for PrPres. Scale bars indicate 200 micrometers.

The present results indicate that white fat and brown fat are possible tissue sources of prion infectivity which might play a role in transmission of prion disease. In vivo brown fat has a limited distribution, usually found in young animals in the intrascapular region and around various organs such as heart and kidney. In adult ruminants brown fat is minimal. Therefore, brown fat from infected animals is unlikely to be consumed by humans in large amounts. In contrast, humans often consume large amounts of ruminant white fat. In premium cuts of meat containing mostly skeletal muscle, white fat is often intertwined with muscle cells, and it is impossible to separate the two cell types. However, white fat, free of muscle, is found in subcutaneous, retroperitoneal, intraperitoneal, perirenal and other regions. Such fat is used in many processed meat products such as sausages and canned meats, and is also used in animal feeds. Our present data show clearly that fat in the absence of muscle has significant infectivity titers, which are similar to titers in muscle containing fat (Table 1). Since our skeletal muscle samples are unavoidably contaminated by white fat, it is possible that fat might be a contributor to the infectivity found in muscle. In support of this possibility we found PrPres detectable by IHC at high levels in white fat associated with skeletal muscle in some tg44 mice (Figure 4). In contrast, other groups did not mention seeing PrPres in muscle-associated fat tissue in animals where myocytes themselves were seen to be positive by IHC [13]–[20].

It is unclear why there is accumulation of PrPres and infectivity in adipose tissues. One possibility might be the high level of innervation by the autonomic nervous system in both brown and white fat. In WT mice, nerves should express cell membrane anchored PrP^C^ (PrPsen). Sympathetic nerves have been previously implicated in transfer of scrapie infectivity from spleen to brain in mice [29], and they might also play a role in infection of fat in WT mice. In tg44 mice the mechanism of fat infection is likely to be different as there is no anchored PrPsen on the nerves. We currently postulate a role for connective tissue structures in this process.

Infectivity in fat might also contribute to environmental contamination following the death of prion infected animals. Although infectivity titers are lower in fat and muscle than in CNS, the large mass of fat and muscle makes the total infectivity from these sources similar. Furthermore, fat and muscle are readily accessible to the environment after death, whereas the CNS is highly confined in skull and vertebral column. These factors might increase the importance of fat and muscle as sources of spread of prion disease among animals.

The low or negative plasma titers found in tg44 and WT mice indicate that residual plasma cannot account for the high infectivity levels seen in fat and other tissues (Table 1). However, low levels of plasma or blood-borne infectivity might still be a mechanism for spread of infectivity among tissues in tg44 mice and possibly also WT mice. Similarly transmission of low level blood prion infectivity has been documented by blood transfusion in BSE-infected sheep [30], and also accounts for some rare cases of human variant CJD [31],[32].

In this study extraneural infection was much higher in tg44 mice expressing anchorless PrP than in WT mice. The explanation of this finding is unclear. Possibly soluble anchorless PrP facilitates spread of infection from CNS to extraneural sites by blood, lymph or nerve-mediated transport. Alternatively, the long asymptomatic survival time of tg44 mice might also contribute to high level extraneural infection. This could also be a factor in many animal prion diseases where the time course is long, i.e. 2–5 years or more, and might allow higher extraneural infectivity in fat tissues [7], [33]–[35].

The present data using a mouse model shows the proof of principle that brown and white fat tissues can be important sites of prion agent deposition. It will be important to extend these studies in the future to prion infected large animals such as cattle, sheep and cervids where there may be greater potential for contamination of human or domestic animal food chains. We are currently doing this experiment with fat from CWD deer; however, it will require an additional year to gather this data, and this result is therefore beyond the scope of the present paper. Such studies may be difficult because of the lower titers seen in these large animals compared to rodent scrapie models. For example, we often detect titers of 9–10 logID50/gram of mouse brain, whereas in brain from BSE cattle [8], and scrapie sheep [4] titers reported are 7–8 logID50/gram. We are finding similar low titers in CWD cervid brain in our deer PrP transgenic mice (unpublished data). These results could indicate either that the amount of prion agent present in ruminant brain is lower than in mice and hamsters or that the cattle, sheep and deer PrP transgenic mice used for infectivity assays are less sensitive than the WT mice or hamster PrP transgenic mice used for rodent scrapie. In either case this might affect ability to detect infectivity in fat of these important large animal models.

## Materials and Methods

### Experimental Mice and Tissues

All mice were housed at the Rocky Mountain Laboratories (RML) in an AAALAC-accredited facility and experimentation followed NIH RML Animal Care and Use Committee approved protocols. Transgenic GPI anchorless PrP mice (tg44) expressed the transgene in heterozygous form (+/−) and did not express WT mouse PrP (MoPrnp−/−) [24]. Mice were bred and genotyped at RML. Weanling C57BL/10 mice were obtained from Jackson Laboratories, Maine. Four to six week old mice were inoculated with a 1% brain homogenate of 22L or RML scrapie containing 0.7–1.0×10^6^ ID_50/_50 µl. One ID_50_ is the dose causing infection in 50% of WT mice. One hundred µl was used for IP inoculations and 50 µl was used for IC inoculations. Animals were observed daily for onset and progression of scrapie. Tissues were collected from WT mice when they were in an advanced stage of scrapie (150–160 dpi). Tg44 mice did not have obvious clinical signs of disease. Tissues were collected from IC inoculated mice at 482–654 dpi and from IP inoculated mice at 441 and 692dpi. Skeletal muscle was obtained from the quadriceps muscle because one previous report indicated that the lower extremity muscles were preferable for detection of muscle positive for PrPres or infectivity [12]. Brown fat associated with kidney and heart were the original sites where we detected PrPres by IHC. However, to obtain brown fat free of other structures, we took brown fat overlying the back muscles between the scapulae. White fat free of muscle was obtained from both perirenal and subcutaneous regions. Two additional tg44 plasma pools were collected at 365 and 450 dpi. Each pool had an equal volume of plasma from three tg44 mice. For solid tissues a portion of each was flash frozen for future use in biochemical analysis and bioassays. A second aliquot was immersed in 10% neutral buffered formalin for immunohistochemisty studies. All individual tissues were collected using new, sterile instruments to prevent cross contamination between tissues. Twenty percent (w/v) tissue homogenates were prepared in 0.01 M Tris buffer pH 7.4 using a disposable mortar and pestle (Kontes, Vineland, NJ), tissue homogenizer (OMNI International TH, Marietta, GA) or mini-beadbeater (Biospec products, Bartlesville, OK). All homogenates were sonicated for 60 sec using a Vibracell cup-horn sonicator (Sonics, Newtown, NJ), vortexed and then frozen.

### Animal Bioassay

End-point tissue titrations were carried out using homogenates of infected tissues from WT and tg44 mice. All titrations were done in C57BL/10 recipient mice from Jackson Laboratories. Additionally, some samples were also titered in tga20 mice [36], which have a higher PrP expression level and faster scrapie incubation periods. Although incubation periods were shorter in tga20 mice compared to C57BL/10 mice, end-point titers were similar (±0.5 log_10_ID50), and values from both end-points were averaged.

Tissue inocula were made in PBBS+2% FBS by preparing serial ten-fold dilutions of a 1% ( = 10^−2^ dilution) tissue homogenate out to 10^−8^ or 10^−10^. Plasma samples were prepared beginning with either a 1% or 10% dilution and tested out to 10^−5^. Fifty µl of each dilution was inoculated intracerebrally into 6–12 mice. Mice were observed daily for onset of scrapie and euthanized when signs such as ataxia, somnolence, kyphosis, and weakness with rear leg clasping reflex were present. Scrapie infection was confirmed by western blot analysis of brain homogenates. Mice that did not become clinically sick were monitored a minimum of 600 days. Healthy mice at this point were considered uninfected for purposes of determining the tissue titer. Titers were calculated using the Spearman/Karber formula [37]. The final value reported was adjusted to show the log_10_ID50/gram or ml of tissue.

### Immunoblotting

Tissue sets from infected WT and tg44 mice and brains from bioassay recipients were analyzed for the presence of PrPres by immunoblot. PrPres preparation was done as previously described [38]. Briefly, 20 µl of a 20% tissue homogenate was adjusted to 100 mM Tris HCl (pH 8.3), 1% Triton X-100, and 1% sodium deoxycholate in a total volume of 31 µl. Samples were treated with 50 µg/ml of proteinase K for 45 minutes at 37°C. The reaction was stopped by adding 2 µl of 0.1 M phenylmethylsulfonyl fluoride and placed on ice for 5 min. An equal volume of 2× Laemmli sample buffer (Biorad, Hercules, CA) was added, and then tubes were boiled 5 minutes. Samples were frozen at −20 until electrophoresed on a 16% SDS-PAGE gel (Invitrogen, CA). Immunoblots were probed with D13 anti-PrP antibody (InPro Biotechnology, Inc., S. San Francisco, CA) followed by a peroxidase-conjugated anti-human IgG secondary antibody (Sigma, St. Louis, MO). Bands were detected using enhanced chemiluminescence substrate (ECL) as directed (Amersham, now GE Healthcare).

### Immunohistochemistry

Tissues were removed and placed in 3.7% phosphate-buffered formalin for 3 to 5 days before dehydration and embedding in paraffin. Serial 4 µm sections were cut using a standard Leica microtome, placed on positively charged glass slides and dried overnight at 56° C. For PrPres detection slides were rehydrated in 0.1M citrate buffer, pH 6.0 and then heated at 120° C, 22psi for 20m in a decloaking chamber (Biocare, Walnut Creek, CA). Immunohistochemical staining was performed using the Ventana automated Nexus stainer (Ventana, Tucson, AZ). Staining for PrP used a standard avidin-biotin complex immunoperoxidase protocol using anti-PrP antibody D13 at a dilution of 1∶500–1∶1500 (In-Pro Biotechnology), biotinylated anti-human IgG at 1∶500 (Jackson Laboratories), and avidin-horseradish peroxidase followed by amino-ethyl carbazole (Ventana). Amyloid staining was done using a five minute immersion in 1.0% w/v of Thioflavin S (MP Biomedicals). Slides were rinsed in 80% EtOH (3x) and water (1x) prior to mounting and coverslipping. The staining of PrPres and amyloid was observed using an Olympus BX51 microscope and Microsuite FIVE software.
